# Editorial: The repercussions of maternal inflammation in pre-eclampsia on fetal health and neurodevelopment

**DOI:** 10.3389/fimmu.2024.1434260

**Published:** 2024-05-29

**Authors:** Igor V. Lakhno, José Javier Reyes-Lagos, Ishag Adam, Fiona C. Brownfoot

**Affiliations:** ^1^ Kharkiv National Medical University, Kharkiv, Ukraine; ^2^ Universidad Autónoma del Estado de México, Toluca, México, Mexico; ^3^ Unaizah College of Medicine, Qassim University, Unaizah, Saudi Arabia; ^4^ The University of Melbourne, Parkville, VIC, Australia

**Keywords:** Inflammation, pre-eclampsia, autonomic nervous system, heart rate variability, neuroinflammation, HLA incompatibility

Inflammation is a crucial and inevitable biological response in humans, playing an essential role in defense against infections and tissue repair. It is known as a basic pathological process. Inflammatory response is involved in the pathogenic scenario of several obstetrical syndromes. The main trigger for maternal inflammation is a disturbed placentation. HLA (Human Leukocyte Antigen) incompatibility between maternal and fetal tissues significantly influences the risk of preterm birth and pre-eclampsia (1). Therefore, immune-induced inflammation is a part of pre-eclampsia.

C. Redman et al. found that pre-eclampsia featured increased levels of pro-inflammatory cytokines (2). The theory of neuroinflammation explains the participation of the autonomic nervous system (ANS) in the induction and evolution of phlogistic reactions (3). The sympathetic activity modulates inflammation by enhancing its intensity. On the contrary, vagal tone demonstrated anti-inflammatory activity (4).

Pregnancy is associated with biphasic changes in autonomic regulation. The elevated parasympathetic tone in the first half of pregnancy captures a gestational resetting. It provides gestational hypervolemia and decreased vascular tone. The increased power of sympatho-adrenal regulation is typical for the second half of a healthy pregnancy. Pre-eclampsia up-regulates sympathetic tone, disturbing prior autonomic balance (5).

Heart rate variability (HRV) is a convenient tool for assessing ANS activity. A coupling between maternal and fetal HRV variables has been proposed. Relationships between maternal and fetal linear and non-linear variables, which are responsible for sympathetic and parasympathetic regulation, have been identified (6). Another study demonstrated the relationship between maternal and fetal very low-frequency domain power spectrum density (7). These findings showed the involvement of maternal autonomic and endocrine regulation in fetal autonomic response. The relations between maternal and fetal HRV are reduced in pre-eclamptic patients (8). Pre-eclampsia is involved in the fetal programming of diseases, causing delayed neurological maturation (9). Since the maternal autonomic malfunction demonstrates pro-inflammatory changes in pre-eclampsia, the disturbed fetal autonomic tone was secondary to maternal inflammation.

The Research Topic was focused on the elucidating role of maternal inflammation in fetal neurological maldevelopment in pre-eclampsia.

On this Research Topic, Pichardo-Carmona et al., reported data on cardiorespiratory coupling in pre-eclamptic patients during labor. The relation between hemodynamics and the respiratory system is critically dependent on vagal-mediated sinus respiratory arrhythmia (SRA). For the detection of cardiorespiratory synchronization, the non-linear methods based on information theory, such as mutual information (MI), Renyi’s mutual information (RMI), and the Pulse-Respiration Quotient (PRQ) were used. The MI and RMI values were significantly lower (p<0.05) in the preeclamptic groups compared to the control group. Additionally, a robust time-domain measure of RSA (logRSA) and normalized permutation entropy of PRQ were significantly lower (p<0.05) in severe pre-eclampsia compared to control and mild pre-eclampsia. The study presented novel evidence on cardiac and respiratory interactions quantifying the cardiorespiratory coupling by nonlinear methods based on information theory and assessing PRQ time series in parturient women with a clinical diagnosis of preeclampsia. The potential dysregulation in the maternal cholinergic anti-inflammatory pathway may be reflected as a decreased cardiorespiratory coupling with concomitant vagal withdrawal.

Stefańska et al. explored the role of maternal-fetal HLA incompatibility in the pathogenesis of gestational hypertension and its progression to pre-eclampsia. The immunogenicity of discordant HLA antigens is determined by functional epitopes called eplets, which are continuous and discontinuous short sequences of amino acids. High-resolution next-generation sequencing of HLA-A, -B, -C, -DQB1, and -DRB1 antigens was performed in mothers and children from physiological pregnancies and from pregnancies complicated with gestational hypertension (22 pairs) and preeclampsia. In the next step HLA eplet identification and analysis of HLA eplet incompatibilities was performed. Simultaneously maternal sera were screened for anti-fetal HLA class I, class II and anti-MICA antibodies with Luminex, and data were analyzed with HLA-Fusion software. The authors observed that high HLA-C, -B, and DQB1 maternal-fetal eplet compatibility was associated with severe preeclampsia manifestation. Both quantity and quality of HLA epletmismatches affected the severity of PE. High HLA-C, HLA-DQB1 and HLA-B eplet compatibility between mother and child is associated with severe preeclampsia. Both quantity and quality of maternal-fetal HLA eplet mismatches affects severity of preeclampsia.

In the perspective article by Abarca-Castro et al., maternal immune activation was proposed to be linked to the pathogenesis of pre-eclampsia and adverse neurodevelopmental outcomes in the offspring, such as cognitive deficits, behavioral abnormalities, and mental disorders. The cholinergic anti-inflammatory pathway (CAP) may play a relevant role in regulating the maternal inflammatory response during pre-eclampsia and protecting the developing fetus from inflammation-induced damage. Dysregulation in the CAP has been associated with the clinical evolution of pre-eclampsia. Some studies suggest that therapeutic stimulation of this pathway may improve maternal and fetal outcomes in preclinical models of pre-eclampsia. Modulation of vagal activity influences the CAP, improving maternal hemodynamics, limiting the inflammatory response, and promoting the growth of new neurons, which enhances synaptic plasticity and improves fetal neurodevelopment. Therefore, the authors postulate that modulation of vagal activity may improve maternal and fetal outcomes in pre-eclampsia by targeting underlying immune dysregulation and promoting better fetal neurodevelopment. In this perspective, the authors explore the clinical and experimental evidence of electrical, pharmacological, physical, and biological stimulation mechanisms capable of inducing therapeutical CAP, which may be applied in pre-eclampsia to improve the mother’s and offspring’s quality of life.

Lucero et al. investigated the fetal hemodynamic oscillations during the latent phase of labor. Linear and nonlinear features of beat-to-beat fHRV, including temporal, frequency, symbolic dynamics, and entropy measures, were analyzed to compare normotensive and preeclamptic groups. Significantly lower values of multiscale entropy (MSE) and short-term complexity index (Ci) were observed in the preeclamptic groups compared to the C group (p < 0.05). Additionally, higher values of SDNN (standard deviation of R-R intervals) and higher values of LF (low frequency) were found in the P group compared to the C group. Their findings indicate that changes in the complexity of fetal heart rate fluctuations may indicate possible disruptions in the autonomic nervous system of fetuses in groups affected by undiagnosed preeclampsia during pregnancy. Reduced complexity and shifts in fetal autonomic cardiac activity could be associated with preeclampsia’s pathophysiological mechanisms during the latent phase of labor.

The collection of articles in this Research Topic underscores the intricate link between maternal inflammation in pre-eclampsia and its repercussions on fetal health and neurodevelopment. Together, these studies advance our understanding of the maternal-fetal interface in pre-eclampsia and highlight potential therapeutic avenues to mitigate adverse outcomes.

## Author contributions

IL: Writing – review & editing, Writing – original draft. JR-L: Writing – review & editing, Writing – original draft. IA: Writing – review & editing. FB: Writing – review & editing.
